# Low-force human–human hand interactions induce gait changes through sensorimotor engagement instead of direct mechanical effects

**DOI:** 10.1038/s41598-024-53991-4

**Published:** 2024-02-13

**Authors:** Mengnan Wu, Madeleine E. Hackney, Lena H. Ting

**Affiliations:** 1 The Wallace H. Coulter Department of Biomedical Engineering, Emory University and Georgia Institute of Technology, Atlanta, GA USA; 2 Division of Geriatrics and Gerontology, Department of Medicine, Emory University School of Medicine, Atlanta, GA USA; 3 Division of Physical Therapy, Department of Rehabilitation Medicine, Emory University School of Medicine, Atlanta, GA USA

**Keywords:** Motor control, Sensorimotor processing, Translational research, Biomedical engineering

## Abstract

Physical human–robot interactions (pHRI) often provide mechanical force and power to aid walking without requiring voluntary effort from the human. Alternatively, principles of physical human–human interactions (pHHI) can inspire pHRI that aids walking by engaging human sensorimotor processes. We hypothesize that low-force pHHI can intuitively induce a person to alter their walking through haptic communication. In our experiment, an expert partner dancer influenced novice participants to alter step frequency solely through hand interactions. Without prior instruction, training, or knowledge of the expert’s goal, novices decreased step frequency 29% and increased step frequency 18% based on low forces (< 20 N) at the hand. Power transfer at the hands was 3–700 × smaller than what is necessary to propel locomotion, suggesting that hand interactions did not mechanically constrain the novice’s gait. Instead, the sign/direction of hand forces and power may communicate information about how to alter walking. Finally, the expert modulated her arm effective dynamics to match that of each novice, suggesting a bidirectional haptic communication strategy for pHRI that adapts to the human. Our results provide a framework for developing pHRI at the hand that may be applicable to assistive technology and physical rehabilitation, human-robot manufacturing, physical education, and recreation.

## Introduction

How best to design powered devices to interact with the human sensorimotor system for assistance and rehabilitation remains a challenge. The substantial mechanical power provided by robotic devices might cause the user to rely more on the device and less on their own abilities^1^. Current physical human–robot interaction (pHRI) approaches to aiding and improving human walking rely primarily on mechanical effects. Lower-limb exoskeletons and robotic gait trainers exert forces directly on parts of the body that propel locomotion. Thus, wearable robotic devices may not require voluntary effort from the user to walk, limiting efficacy for rehabilitation^2,3^. Hand-contact robotic walkers also rely on mechanical effects as they are often designed to support substantial bodyweight^4–7^.

In contrast, we take an alternative approach to pHRI that induces the person receiving aid to engage sensorimotor processes to alter their own gait. Our approach is inspired by examples of low-force physical human–human interaction (pHHI) at the hands during walking. We define “low” magnitude hand interaction force as within the range used by human pairs to perform non-mechanical tasks, e.g. ≤ 30 N for a handshake^8^ and communicating walking transitions^9^. This range is higher than the ≤ 3 N range observed for hand contact during walking without an explicit task^10^. Using low-force hand interactions to influence changes to gait has the potential to mechanically decouple the site of physical interaction—the hands—from the site of targeted motor behavior—the lower limbs, requiring human perceptual and motor systems to interpret and respond to physical interactions. Humans have amassed experiences from everyday activities such as one person holding another’s hand to aid balance or signal turning direction during walking. As such, low-force hand interactions to aid walking may also be intuitive, i.e. require minimal user training and no explicit instructions. We seek to take advantage of existing human sensorimotor mappings between hand interactions and walking to make pHRI devices that are intuitive for people to use.

Few pHRI studies have examined the use of low-force hand interactions to influence human gait, and few pHHI studies have provided principles of how gait changes occur. Two pHRI studies showed that robotic devices applying constant tensile forces of 5 N^11^ or 10–20 N^12,13^ on a person’s hand in the forward direction result in increased gait speed and step length^11–13^. However, it is unknown whether changes to gait in these studies were due to engagement of sensorimotor processing or passive mechanical effects of pulling the body forward. Furthermore, these previous pHRI studies did not isolate the effects of hand interactions, as audio-visual feedback was simultaneously used. Meanwhile, pHHI studies have shown that low-force (≤ 30 N) hand interactions can result in a variety of changes to walking, including synchronization of gait phase^10,14^, communication of walking transitions^9^, and improved balance during walking^15^. However, these pHHI studies have not characterized human control strategies in a manner applicable to design of pHRI.

Partner dancing provides an exemplar framework for examining how low-force human–human hand interactions can affect walking but has been little studied in a controlled experimental paradigm. Partner dancing has formed the basis for rehabilitation interventions that improve walking speed and dynamic balance in impaired populations^16,17^. However, only one previous laboratory study collected motion capture and hand interaction force data during a partnered stepping task^9^. The study showed that low hand forces can communicate information on desired walking behavior unidirectionally, from the person walking forwards to the person walking backwards^9^. Unfortunately, that paradigm does not readily translate to pHRI devices that rely on human sensorimotor engagement, where the person ideally sends *and* receives information to/from the robot about desired walking. A novel bidirectional approach for influencing walking is provided by the partner dancing paradigm of “backleading,” where the person walking backwards both responds to and exerts influence on the person walking forwards^18^.

In the current study, an expert backleader uses hand interactions to influence novices to increase or decrease their own step frequency, a gait parameter not targeted through hand interactions in previous studies. Our central hypothesis is that low-force hand interactions in human pairs induce gait changes intuitively without relying on mechanical effects. First, we validate the efficacy and intuitiveness of our backleading paradigm by testing for intended changes to novices’ step frequency without explicit instructions or training. We predict that hand interaction forces are low (i.e. within the range used by human pairs for non-mechanical purposes), and mechanical power transfer at the hands is substantially lower than that of the lower limbs on the center-of-mass to propel locomotion. To characterize control strategies used by the partnership and each partner, we examine spatiotemporal metrics of the partners’ torsos and arms. Finally, to glean principles from pHHI applicable to pHRI, we develop a “template”^19^ model of hand interactions that represents a system’s total effective dynamics, which includes the combined effects of closed-loop neural commands, muscle activation dynamics, and mechanical impedance. Our backleading paradigm allows us to model the physical interaction decoupled from the primary motor task of walking. Specifically, we predict that the expert modulates her arm effective dynamics in order to communicate walking goals to different novices through hand interactions.

## Results

**Figure 1:**
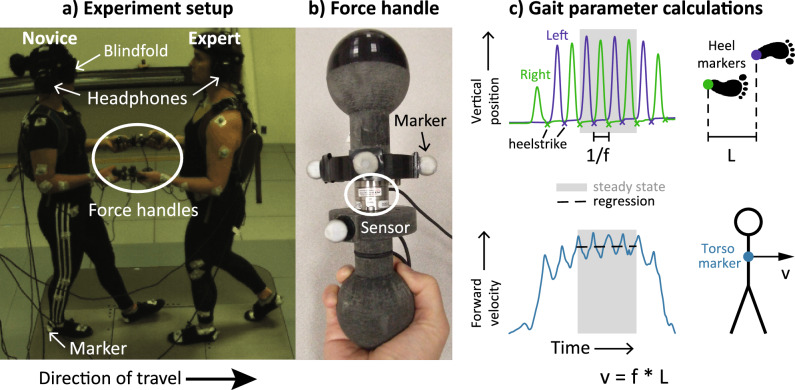
Partnered human–human backleading experiment setup and gait parameter calculations. (**a**) Experiment setup. During backleading conditions, novice participants walked forwards while the expert walked backwards; both partners held opposite ends of custom force handles. Audiovisual cues to the novice were masked. The expert listened to audio instructions and cues at the desired step frequency. Whole-body motion capture and hand interaction force data were collected. (**b**) Close-up of custom force handle. (**c**) Gait parameter calculations. The novice’s step frequency (f) and step length (L) were calculated using foot marker data. The novice’s gait velocity (v) was calculated using the torso marker. The period from the second to fourth right heelstrikes was checked for steady state by testing for zero regression slope of torso velocity. Mean values for f, L, and v were calculated during the steady state period.

We collected data on 10 young adults who walked with a single expert backleader (Fig. 1, Supplementary video at https://osf.io/pzvws). Both partners held opposite ends of force sensors, and novices were instructed to maintain a constant arm posture. The novice walked forwards while the expert walked backwards. All participants completed 3 backleading conditions in a within-subjects study design. The order of presentation of conditions was randomized. Only the expert received information on the goal of each condition—to Decrease, Maintain, or Increase the novice’s step frequency relative to the novice’s preferred step frequency during solo walking. We examined full-body kinematics and hand interaction forces during the period of steady-state walking for each trial. Our results show intended changes to step frequency and low (< 20 N) hand forces across backleading conditions.

For each trial, the novice was instructed to walk forward 8 steps and collect the feet at the 9th step, starting with the right foot and ending with both feet side-by-side. To demonstrate that hand interactions intuitively influence walking, novices did not receive explicit instructions or training on how to walk and were given only one or two practice trials walking solo and with a partner.

### Novice gait parameters

**Figure 2:**
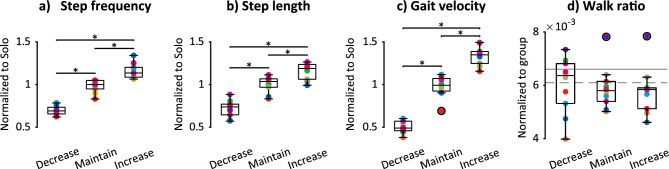
Novice gait parameters across backleading conditions. (**a**) Step frequency, (**b**) step length, and (**c**) gait velocity across conditions, normalized to individual solo walking values. (**d**) Walk ratio normalized according to methods in previous work^20^. (**a**–**d**) Boxplots show group medians, interquartile range, and outliers (black circles). Colors denote each novice participant (n = 10). *Significantly different post-hoc comparisons. (**d**) Gray lines show mean solo walk ratios for female (solid) and male (dashed) young adults from previous work^20^.

The Increase/Decrease backleading conditions caused the intended ± 20% changes to the novice’s step frequency (Fig. 2a) along with similar changes to step length and gait velocity (Fig. 2b,c). Across participants, step frequency decreased by ~ 29% and increased by ~ 18% in Decrease and Increase conditions, respectively, relative to the Maintain condition (Fig. 2a). Step length decreased by ~ 28% and increased by ~ 16% in Decrease and Increase conditions, respectively, relative to Maintain (Fig. 2b). Gait velocity decreased by ~ 49% and increased by ~ 37% in Decrease and Increase conditions, respectively, relative to Maintain (Fig. 2c). Accordingly, backleading condition had a significant effect on step frequency (F_2,18_ = 221.6, p < 0.001), step length (F_1.3,11.6_ = 199.6, p < 0.001), and gait velocity (F_2,18_ = 425.3, p < 0.001) in repeated measures ANOVAs. All condition means were significantly different from each other in post-hoc comparisons (all p < 0.001) for step frequency, step length, and gait velocity (Fig. 2a–c).

A measure of gait coordination, the walk ratio^20–22^—the ratio between step length and step frequency—remained constant across conditions. Walk ratio was previously shown to remain constant across speeds in unconstrained walking^20–22^. Accordingly there was no effect of backleading condition on the walk ratio (repeated measures ANOVA: F_1.1,9.8_ = 0.6, p = 0.470), with condition means similar to values previously measured in unimpaired young adults^20^ (Fig. 2d).

### Signed force, velocity, and power

**Figure 3:**
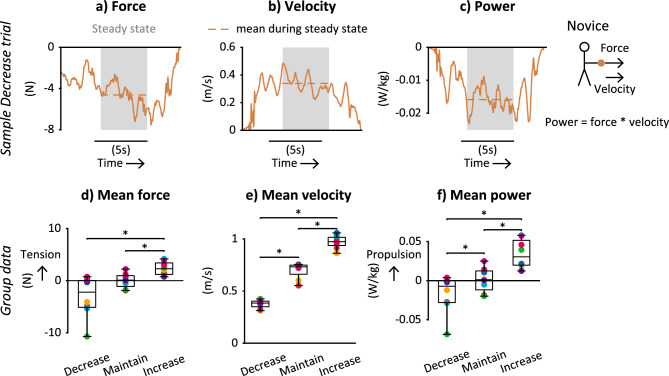
Signed interaction point force, velocity, and power across backleading conditions. Example traces of (**a**) force, (**b**) velocity, and (**c**) power at the hand from a Decrease trial for one partnership. (**d**) Mean force across participants in each condition. Positive values correspond to tension; negative values correspond to compression. (**e**) Mean velocity across participants in each condition. Positive values correspond to the forward direction. (**f**) Mean power across participants in each condition. Positive values correspond to propulsion; negative values correspond to braking. (**d**–**f**) Boxplots show group medians, interquartile range, and outliers (black circles). Colors denote each partnership (n = 8). *Significantly different post-hoc comparisons.

The signs of mean interaction force and power at the hand corresponded to the direction (increase or decrease) of the desired changes to step frequency (Fig. 3). Time histories of force, velocity, and power at the hand (example traces in Fig. 3a–c) fluctuated during each trial, ramping up or down at the beginning and end of each trial. We analyzed the middle 4 steps of each trial, when these variables reached an approximate steady state, fluctuating around a mean value. Backleading condition had a significant effect on mean signed force (F_1.1,7.4_ = 11.9, p = 0.009), velocity (F_2,18_ = 416.7, p < 0.001), and power (F_1.1,7.7_ = 18.3, p = 0.003) in repeated measures ANOVAs.

Across participants, interactions forces (Fig. 3d) were in compression during Decrease (− 3.07 ± 3.95 N), zero during Maintain (0.001 ± 0.016), and in tension during Increase (2.34 ± 1.27) conditions. Mean force during Increase was significantly different from both Decrease (p = 0.028) and Maintain (p = 0.014) conditions, respectively, in post-hoc comparisons (Fig. 3d).

Across participants, interaction point velocities (Fig. 3e) were different across all conditions (p < 0.001 in all post-hoc comparisons), with velocities of 0.37 ± 0.05 m/s in Decrease, 0.71 ± 0.09 m/s in Maintain, and 0.98 ± 0.08 m/s in Increase.

The signs of power (Fig. 3f) were consistent with absorbing energy or braking during Decrease (− 0.017 ± 0.024 W/kg) and injecting energy or propulsion during Increase (0.034 ± 0.018 W/kg) conditions. All condition means were significantly different from  each other in post-hoc comparisons (p = 0.032 for Decrease vs. Maintain, p = 0.010 for Decrease vs. Increase, and p = 0.009 for Maintain vs. Increase).

### Force and power magnitudes

**Figure 4:**
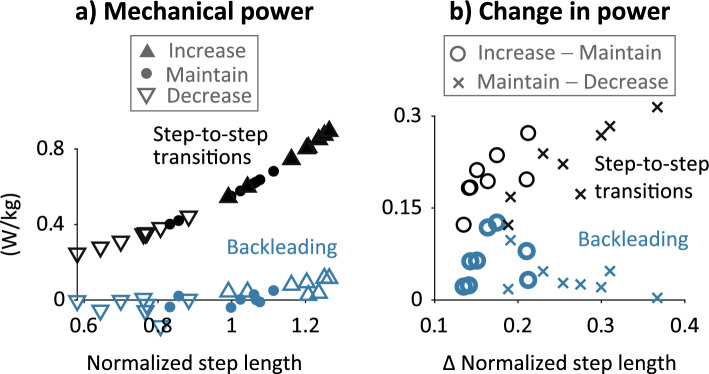
Mean mechanical power measured during backleading compared to estimated power for step-to-step transitions during solo walking. Power for backleading (blue markers) was measured at one hand and multiplied by two for both hands. Power for step-to-step transitions (black markers) was calculated at mean step lengths for each participant and backleading condition using the equation in Donelan et al.^23^. (**a**) Mean power vs. step length for each condition (triangle and circle symbols). 6 data points per novice participant (n = 8). (**b**) Change in mean power between conditions (“x” and “o” symbols). 4 data points per novice participant (n = 8).

Hand interaction forces and power magnitudes were relatively small across all backleading conditions. Maximum force and power did not exceed 20 N and 0.15 W/kg, respectively, during steady-state walking for any partnership or condition (Supplementary Fig. S1: https://osf.io/6cxwe). Mechanical power transfer for both hands during backleading was ~ 3–700 × smaller than estimated power for step-to-step transitions during solo walking, computed using the method of Donelan et al.^23^ (Fig. 4). Mean hand interaction power magnitudes ranged 0.009–0.137 W/kg across participants and backleading conditions, whereas estimated step-to-step transition power magnitudes at equivalent step lengths ranged 0.248–0.894 W/kg (Fig. 4a). Magnitudes of  changes in mean hand power between conditions ranged 0.004–0.130 W/kg, whereas magnitudes of changes in estimated step-to-step transition power for equivalent changes in step lengths ranged 0.122–0.315 W/kg (Fig. 4b).

### Spatiotemporal metrics characterizing control strategies

We examined several spatiotemporal metrics to characterize how the partnership coordinated gross body movement with each other and how each partner contributed to inter-partner coordination.

**Figure 5:**
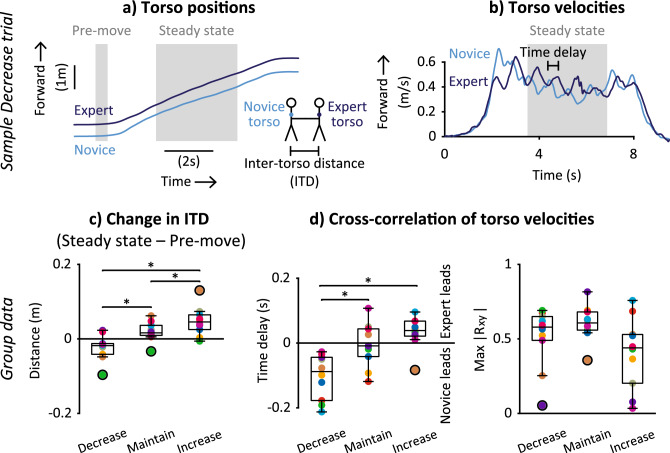
Spatiotemporal synchrony between partners’ torsos across backleading conditions. Example time-series data of (**a**) torso positions and (**b**) torso velocities from a Decrease trial for one partnership. (**c**) Change in mean inter-torso distance (ITD) from pre-move to steady-state periods. (**d**) Time delay and maximum absolute cross-correlation (max |R_xy_|) of partners' torso velocities. (**c**,**d**) Boxplots show group medians, interquartile range, and outliers (black circles). Colors denote each partnership (n = 10). *Significantly different post-hoc comparisons.

Spatially, partners were closer together in the Decrease condition and further apart in the Increase condition (Fig. 5). The signs of Change in Inter-torso distance (ITD), measured using trunk markers, show that, relative to the period of standing preceding walking (“pre-move” in Fig. 5a), distance between partners’ torsos during steady-state walking were smaller (Change in ITD ~ − 0.02 m) in the Decrease condition and larger (Change in ITD ~ 0.05 m) in the Increase condition (Fig. 5c). Accordingly, backleading condition had a significant effect on Change in ITD in repeated measures ANOVA (F_1.2,10.7_ = 20.9, p = 0.001). All condition means for Change in ITD were significantly different from each other in post-hoc comparisons: Decrease < Maintain (p = 0.005), Decrease < Increase (p = 0.003), and Maintain < Increase (p = 0.010) (Fig. 5c).

The novice's torso motion led that of the expert during the Decrease condition, whereas the expert’s torso motion led during the Increase condition (Fig. 5). Time delays were identified through cross-correlation analysis of torso velocities during the steady-state period (Fig. 5b). The signs of the time delay show that the novice’s torso motion preceded the expert’s torso motion (time delay ~ − 0.10 s) in the Decrease condition, and the expert’s torso motion preceded the novice’s torso motion (time delay ~ 0.03 s) in the Increase condition (Fig. 5d, left). Accordingly, backleading condition had a significant effect on time delay between torso velocities in repeated measures ANOVA (F_2,18_ = 12.9, p < 0.001). Mean time delay in Decrease was significantly different from both Maintain (p = 0.002) and Increase (p = 0.006) conditions in post-hoc comparisons (Fig. 5d, left). Backleading condition did not have a significant effect on maximum absolute cross-correlation between torso velocities in repeated measures ANOVA (F_2,18_ = 2.9, p = 0.080; Fig. 5d, right).

**Figure 6:**
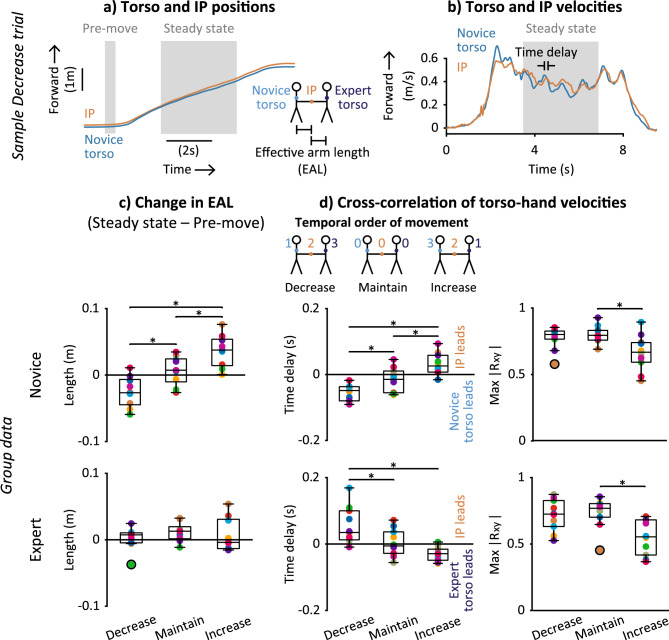
Spatiotemporal synchrony between interaction point and each partner’s torso across backleading conditions. Example time-series data of anterior–posterior torso and interaction point (**a**) positions and (**b**) velocities during from a Decrease trial for one partnership. (**c**) Change in Mean Effective Arm Length (EAL) from pre-move to steady-state periods for the novice (top row) and expert (bottom row). (**d**) Summary of temporal order of movement (top). Time delay and maximum absolute cross-correlation (max |R_xy_|) of torso and interaction point velocities for novice (top) and expert (bottom). (**c**,**d**) Boxplots show group medians, interquartile range, and outliers (black circles). Colors denote each partnership (n = 10). *Significantly different post-hoc comparisons.

Effective arm length (EAL), the distance between the torso and the interaction point at the hand, of the novice was shorter in the Decrease condition and longer in the Increase condition (Fig. 6a,c). The signs of condition means show that, relative to the pre-move period (Fig. 6a), the novice’s EAL during steady-state walking decreased (Change in EAL ~ − 0.025 m) in the Decrease condition and increased (Change in EAL ~ 0.037 m) in the Increase condition (Fig. 6c, top). Accordingly, backleading condition had a significant effect on the novice’s Change in EAL in repeated measures ANOVA (F_2,16_ = 44.0, p < 0.001). All condition means for Change in EAL were significantly different from each other in post-hoc comparisons: Decrease < Maintain (p = 0.006), Decrease < Increase (p < 0.001), and Maintain < Increase (p < 0.001) (Fig. 6c, top).

In contrast to the novice, the expert maintained a constant arm length across backleading conditions (Fig. 6c, bottom). There was no significant effect of backleading condition on the expert’s Change in EAL in repeated measures ANOVA (F_1.2,9.6_ = 0.9, p = 0.383).

Opposite changes in the novice's and expert’s time delays between torso and hand velocities across backleading conditions resulted in reverse temporal orders of movement of partners’ bodies relative to the hand interaction point (Fig. 6b,d). During the Maintain condition, the novice’s torso, the interaction point, and the expert’s torso all moved simultaneously (Fig. 6d). However, changes in time delays of each partner’s torso relative to the interaction point resulted in a reversal of temporal orders of movement when targeting changes to step frequency (Fig. 6d). The temporal orders were (first to last):

- Decrease condition: (1) novice’s torso, (2) interaction point, (3) expert’s torso.
- Increase condition: (1) expert’s torso, (2) interaction point, (3) novice’s torso.

Accordingly, backleading condition had a significant effect on torso-hand time delay in repeated measures ANOVAs (F_2,18_ = 19.0, p < 0.001 for the novice; F_2,18_ = 15.3, p < 0.001 for the expert). All condition means for the novice’s time delay were significantly different from each other in post-hoc comparisons (p = 0.045 for Decrease vs. Maintain, p = 0.001 for Decrease vs. Increase, and p = 0.016 for Maintain vs. Increase; Fig. 6d, top left). The expert’s mean time delay in Decrease was significantly different from both Maintain (p = 0.009) and Increase (p = 0.003) conditions (Fig. 6d, bottom left).

Torso and hand velocities were more correlated in Maintain vs. Increase and similarly correlated in Maintain and Decrease conditions for both partners (Fig. 6d, right column). Accordingly, backleading condition had a significant effect on maximum absolute cross-correlation between torso and interaction point velocities in repeated measures ANOVAs (F_2,18_ = 8.5, p = 0.003 for the novice; F_2,18_ = 6.5, p = 0.008 for the expert). Condition means were significantly different between Maintain and Increase conditions in post-hoc comparisons (p = 0.007 for the novice, p = 0.019 for the expert) (Fig. 6d, right column).

### Models of pHHI dynamics

**Figure 7:**
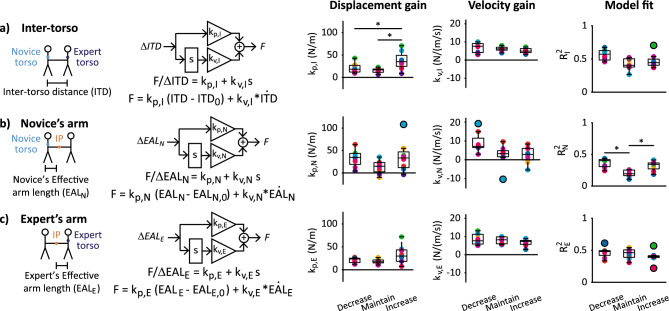
Inter-torso and arm effective dynamics across backleading conditions. Control diagrams, transfer functions, regression equations, and regression coefficients for effective dynamics models describing the relationship between hand interaction force and changes in (**a**) partnership Inter-torso distance (ITD), (**b**) the novice's effective arm length (EAL_N_), or (**c**) the expert's effective arm length (EAL_E_). Reference values ITD_0_, EAL_N,0_, and EAL_E,0_ are calculated during the pre-move period. (**a**–**c**) Control diagrams and transfer functions: “s” is the first-order time derivative; “k_p_” and “k_v_” are displacement and velocity coefficients, respectively; and “F” is hand interaction force. Boxplots show group medians, interquartile range, and outliers (black circles). Colors denote each (**c**) partnership, (**d**) novice participant, or (**e**) expert when partnered with each novice; n = 8 novices/partnerships. *Significantly different post-hoc comparisons.

To glean principles from pHHI applicable to pHRI, we modeled the effective dynamics—the overall relationship between measured kinematics and force—of the partnership and each individual (Fig. 7). Our lumped-parameter models combine the effects of neural control, muscle activation dynamics, and passive mechanics of the body/limbs into a simple description of how hand interaction forces are regulated based on changes to Inter-torso distance (ITD) or Effective arm length (EAL).

The inter-torso effective dynamics model had a higher displacement coefficient (k_p,I_) in Increase compared to Maintain and Decrease conditions (Fig. 7a). Backleading condition had a significant effect on k_p,I_ (F_2,14_ = 11.1, p = 0.001) and model fit (R_I_^2^) (F_2,14_ = 5.7, p = 0.015) but not velocity coefficient (k_v,I_) (F_1.1,7.6_ = 5.1, p = 0.053) in repeated measures ANOVAs. Mean k_p,I_ in the Increase condition was ~ 78% and ~ 143% higher than in Decrease and Maintain conditions, respectively (Fig. 7a); these differences were significant in post-hoc comparisons: Increase > Decrease (p = 0.042) and Increase > Maintain (p = 0.022). R_I_^2^ was not significantly different between conditions in any post-hoc comparisons (p = 0.085, 0.237, 0.349 for Decrease vs. Maintain, Decrease vs. Increase, and Maintain vs. Increase, respectively).

While not significant, each partner’s arm effective dynamics followed similar trends as the inter-torso effective dynamics across conditions (compare across rows in Fig. 7). The novice arm model’s displacement coefficient (k_p,N_) ranged − 10.1 to 108 N/m across conditions, and the expert arm model’s displacement coefficient (k_p,E_) ranged 7.37 to 71.8 N/m. The novice arm model’s velocity coefficient (k_v,N_) ranged − 10.4 to 19.2 N/(m/s) across conditions and the expert arm model’s velocity coefficient (k_v,E_) ranged 2.84 to 13.1 N/(m/s). Backleading condition had no significant effect on k_p,N_ (F_2,14_ = 2.7, p = 0.100), k_v,N_ (F_1.2,8.3_ = 4.2, p = 0.070), k_p,E_ (F_1,7.3_ = 2.4, p = 0.107), or k_v,E_ (F_1.2,8.1_ = 2.9, p = 0.123) in repeated measures ANOVAs.

The novice arm model fit (R_N_^2^) was worse in Maintain compared to the Decrease and Increase step frequency conditions (Fig. 7b). Accordingly, backleading condition had a significant effect on R_N_^2^ in repeated measures ANOVA (F_2,14_ = 14.4, p < 0.001). Mean R_N_^2^ in Maintain was significantly lower than in both Decrease (p = 0.008) and Increase (p = 0.005) conditions in post-hoc comparisons (Fig. 7b).

Backleading condition had no significant effect on the expert arm model fit (R_E_^2^) in repeated measures ANOVA: F_2,14_ = 1.9, p = 0.184 (Fig. 7c).

**Figure 8:**
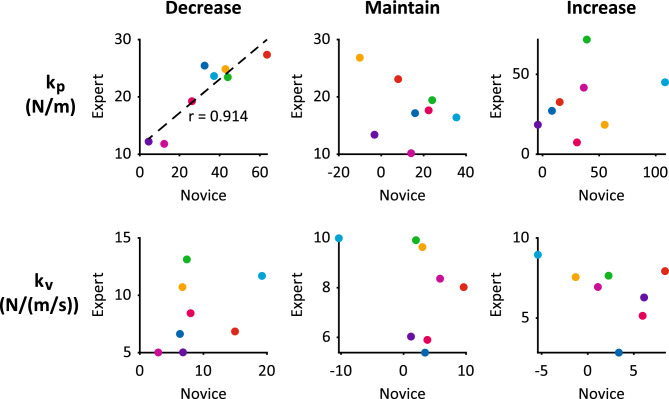
Expert vs. novice arm effective dynamics during each backleading condition. Top row: displacement coefficients (k_p_). Dashed line: significant linear correlation. r: Pearson’s correlation coefficient. Bottom row: velocity coefficients (k_v_). Columns correspond to backleading conditions. Colors denote each novice participant or expert when partnered with each novice; n = 8 novices/partnerships.

The expert’s arm effective dynamics varied according to each novice’s arm effective dynamics in the Decrease condition (Fig. 8, top left). The expert and novice effective arm displacement coefficients (k_p_) were significantly correlated (p = 0.002, r = 0.914) in the Decrease condition but not in Maintain (p = 0.398) or Increase (0.425) conditions, according to Pearson’s correlation (Fig. 8, top row). The expert and novice effective arm velocity coefficients (k_v_) were not significantly correlated in any conditions (Fig. 8, bottom row): Spearman’s p = 0.132 (Decrease), Spearman’s p = 0.268 (Maintain), and Pearson’s p = 0.311 (Increase).

## Discussion

This is the first study to demonstrate the feasibility of using low-force hand interactions to influence human gait parameters in a systematic and intuitive manner without relying on mechanical effects. The efficacy and intuitiveness of the paradigm are demonstrated by observations of intended step frequency changes in novice participants without instructions or training. While we did not constrain hand interaction forces or power, all partnerships used magnitudes much smaller than for mechanically powering changes to gait. We posit that instead of mechanically amplifying or resisting human walking movements, hand interactions can communicate information. Our results suggest that the direction/sign of hand forces and power correspond to desired walking goals, which has ramifications for analysis of emergent roles in dyads. Although we explicitly assigned a dominant role within the partnership to the expert backleader, different task goals could reverse the temporal order of partners’ body movements, which contradicts common assumptions in analysis of dominance/causation in physical human–human interaction (pHHI) studies. Finally, we characterized the effective dynamics of pHHI during backleading to glean principles of how to alter human gait through hand interactions in physical human–robot interaction (pHRI). The results of the current study provide a framework for our long-term vision to develop robot controllers that use low-force hand interactions to aid human walking through sensorimotor engagement.

The efficacy and intuitiveness of our backleading paradigm is demonstrated by the intended changes to the novice’s step frequency, a gait parameter not previously targeted by hand interactions in pHRI or pHHI. Step frequency changes were accompanied by simultaneous changes in step length and gait velocity while maintaining a constant walk ratio, which is consistent with what has been observed previously during self-selected walking in unimpaired persons^20–22^. We also observed larger changes in both gait speed and step length during our Increase condition compared to previous pHRI studies that applied constant tensile forces at the hand in unimpaired young adults. Our backleading paradigm resulted in a 37% increase to gait speed whereas the previous studies showed 25%^11^ and 18–25%^13^ increases, and our paradigm resulted in a 16% increase in step length whereas the previous studies showed 10%^11^ and 11–14%^13^ increases in a similar population. In contrast to previous pHRI studies^11–13^, we did not allow audio or visual information to the human, thereby isolating the effects of hand interactions. However, a potential limitation is that the expert received visual feedback for safety during walking, and thus, it remains unclear whether visual information for the expert is necessary for the paradigm to be effective. The intuitiveness of the paradigm is demonstrated by the fact that gait changes occurred without explicit instructions or training of novices.

The small magnitudes of mechanical power transfer and force at the hands suggest that hand interactions during backleading do not directly cause mechanical changes to the novice’s gait. Power transfer at the hands during backleading was ~ 3–700 × smaller than the power exerted by the lower limbs on the center-of-mass to maintain constant-speed solo walking. Mean hand forces fluctuated around 3 N, similar to that found during handholding during walking without an explicit task^10^ and lower than the 5 N^11^ and 10–20 N forces^12,13^ applied by a robotic device to increase gait speed. Maximum magnitudes remained below 20 N, which is smaller than the 30 N range for a handshake^8^ and communicating walking transitions^9^. Previous studies that perturbed the pelvis or legs in the anterior–posterior direction during walking used forces ranging 4–60% of bodyweight^24–27^; and the smallest forces of ~ 26 N applied at the pelvis did not affect foot placement^27^. Interaction forces between a human and a wearable gait assistance/rehabilitation device are difficult to measure^28,29^ and seldom reported^30^, but physical therapists apply hand forces of 50–100 N to a patient’s leg during gait training^31^. As the expert does not mechanically constrain the novice’s walking, novices must engage sensorimotor processes to alter their own gait.

Low-force hand interactions likely provide information on task goals, such as how to alter step frequency in our current study. We define “haptic communication” as the exchange of information through sensory feedback elicited from physical contact^32^. Haptic communication has been used to explain the benefits of human–human hand interactions for performing several types of upper-limb tasks^33–39^. Our results suggest that the signs of interaction force and power may encode how to alter gait (increase or decrease). As step frequency, step length, and velocity changed similarly directions across backleading conditions, it is not possible to resolve which parameter was the control variable used by the novice or expert. While the signs of force and power are consistent with mechanical processes of compression/tension and braking/propulsion, the small magnitudes are insufficient to mechanically cause the observed changes to gait. Instead, the signs of low-magnitude hand force/power may reflect an intuitive mapping from stimulation of hand skin and/or arm muscle sensory organs to gross body movement. Also consistent with hand interactions serving haptic communication, movement of the hand interaction point occurred temporally between partners’ torso movements, which suggests that hand interaction is a necessary intermediary for coordinating partnered walking. As haptic communication relies on transmission of information, changes in hand force must be detectable by the user for this paradigm to be effective. Future testing in impaired populations is needed, but our results show intended changes to gait even when the novice’s arm posture is not maintained, suggesting that our paradigm may be robust for persons with impaired sensing or control of arm motion.

Our results show that the signs of hand force/power and relative timing of partners’ movements may reflect task goals instead of partners’ roles or direction of causation/influence between partners. Our backleading paradigm fixes bidirectional causation/influence a priori to the interaction: the expert is instructed to both exert influence on and respond to the novice’s movement while the novice is instructed to initiate walking and “cooperate” with the expert. Thus, neither the novice nor expert acts as a pure “leader” or “follower” in any condition. If we applied common methods of inferring emergent—i.e. not fixed a priori—roles and control strategies, we may incorrectly infer leadership roles based on signs/directions of interaction force/torque^33,34,40,41^ and power^42^ or based on which person moved earlier, when in actuality these trends reflect whether the task is to decrease or increase the novice’ step frequency. Other time-domain methods for identifying roles—e.g. calculating time integrals of force data^43,44^—may also be confounded by task effects. When roles are emergent and/or fluid, task context should be considered when inferring roles or directions of causation/influence.

Our modelling results suggest that the expert used dynamics-matching, an example of the principle of *bidirectional* haptic communication, which is applicable to design of pHRI control. Altering her arm effective dynamics to match that of each novice might have facilitated the expert’s ability to communicate information through hand interactions; i.e. the same interaction force maps to a similar arm displacement for both partners, establishing a common “haptic language.” Dynamics-matching in backleading may improve human ability to sense the sign/direction of force/power, which may encode the task goal, the *content* of the haptic communication. Our lumped-parameter “template” models represent the overall effective dynamics of pHHI, which includes neural control commands as well as body/limb mechanical impedance. While our models do not measure mechanical impedance independently, arm effective dynamics modulation to facilitate haptic communication in our study is consistent with results from previous work showing that endpoint mechanical impedance varies systematically during hand interactions that communicate intended movement direction^36,45^. The effective displacement coefficient (k_p_) may be especially crucial for facilitating haptic communication: inter-torso effective displacement coefficient (k_p,I_) increased across backleading conditions, and partners’ arm effective displacement coefficients (k_p,N_ and k_p,E_) were correlated in the Decrease condition. The pHHI principle of bidirectional haptic communication can be implemented in pHRI via robot controllers that adapt based on the human’s state; and the specific pHHI strategy of dynamics-matching may be implemented in pHRI, for example, via an admittance controller that adapts admittance gains based on human arm effective dynamics calculated from measurements of human effective arm length and interaction force.

Our models of inter-partner and individual arm effective dynamics must be interpreted carefully and may require further refinement and development of additional metrics to help interpret model results. In contrast to previous models of pHHI dynamics, our models examine the effective dynamics of physical interaction separate from the dynamics of the targeted motor behavior. Our model has several underlying assumptions—such as linearity and time-invariance—that are not true of biological systems, and more complex models may be necessary in the future. Second-order derivatives of torso/arm kinematics may also be necessary in future models, which would require direct measurements of human torso and hand acceleration that our setup lacked. Finally, our model fits were sometimes low, especially in the Maintain condition, perhaps because targeted step frequency was the same as the novice’s preferred solo step frequency; i.e. the task goal was achieved by default, and little haptic communication was required. To test such a prediction, new techniques are needed to characterize the quantity of information transfer separate from the content of haptic communication.

The sensorimotor-engagement-based approach we examine in this study may encourage humans to rely more on their own abilities than another person or device, improving efficacy for walking rehabilitation and requiring less external power. The novices’ changes to gait based on hand interactions require integration of sensory information from one part of the body to command movement changes in another part of the body. Increased sensorimotor engagement may result in longer-lasting changes to movement than direct mechanical assistance or resistance from an external source. Additionally, wearable robotic exoskeletons and gait trainers^2,3, 46,47^ that directly alter walking mechanics face major technical challenges of large size and weight, whereas our low-force sensorimotor-engagement-based approach may require less electrical power and smaller, lighter actuators and batteries. Low-force hand pHRI may also be used in conjunction with—not instead of—devices that provide propulsion for locomotion to improve gait in impaired persons who are unable to power changes to their own gait.

Our study provides principles of how low-force pHHI at the hands can induce changes to gait that are applicable to design of intuitive, biologically-inspired pHRI controllers. pHRI designed to engage human sensorimotor processes instead of mechanically constraining human movement could pose less risk of injury, consume less electrical power, and be smaller/lighter than existing robotic devices that aid walking. Such low-force pHRI controllers may have wide-ranging applications, including assistive technology and physical rehabilitation (e.g., robotic canes and walkers), human–robot manufacturing (e.g. teleoperation and collaborative load transportation), physical education (e.g. teaching sports and dance), and recreation (e.g. next-generation gaming with  virtual and physical interaction). Greater understanding of what information low-force hand interactions communicate and how this information is communicated has the potential to improve human movement ability, enhance physical collaborations between humans and robots, and facilitate the performance of novel pHRI tasks not previously possible with human partners.

## Methods

The experimental protocol and procedure were approved and in accordance with relevant guidelines and regulations of the Emory University Institutional Review Board (IRB00082414). The participants provided their written informed consent to participate in this study. Informed consent has been obtained to publish the information/image(s) in an online open-access publication.

### Participants

Data collection was completed with ten young adult novices (7 female and 3 male, ages 20 ± 2.3 years, height 1.7 ± 0.12 m, mass 67 ± 14 kg) with no previous partner dancing experience and no diagnosed sensory or motor impairments. The expert paired with each of the ten novices was female, age 46 years, height 1.7 m, mass 67 kg, and had > 20 years of professional teaching experience in partner dancing.

### Experiment setup

We collected full-body motion capture data (n = 10) for both the novice and expert and hand interaction forces (n = 8) between the partners. Each partner was instrumented with a full-body Plug-in Gait marker set (Vicon, Centennial, CO). Kinematic data were recorded at 100 Hz by a ten-camera motion capture system (Vicon Nexus) and force data were recorded at 1 kHz. During partnered walking conditions, each partner held opposite ends of a custom handle device with a force-torque sensor (ATI Nano25) in the center that measured hand interaction forces between the partners (Fig. 1a,b).

### Experiment procedures

Human dyads consisting of the same expert partner dancer paired with different novices participated in a backleading paradigm, where the expert tried to influence novices to alter their own step frequency through hand interactions. We used a within-subjects experiment design to examine changes in the novice’s step frequency across backleading conditions. To test whether the novice changes gait intuitively and if partners use low interaction forces, we did not give instructions on how to walk or interact at the hands.

#### Procedures common to all conditions

To isolate the effects of hand interactions on gait, we masked audiovisual cues of walking. The novice wore a blindfold over both eyes and listened to white noise played through headphones during all walking (Fig. 1a). The expert listened to audio instructions and cues through headphones that blocked ambient sounds. The expert was not blindfolded to ensure safety during walking over several steps.

Throughout the experiment, the novice held a custom force handle (Fig. 1b) in each hand and was instructed to maintain a fixed arm posture with elbows flexed at 90° (Fig. 1a). Maintaining a consistent arm posture has been shown to be important for communicating information through hand interactions during partnered stepping^9^.

For each trial, the novice was instructed to walk forward 8 steps and collect the feet at the 9th step, starting with the right foot and ending with both feet side-by-side. To demonstrate that hand interactions intuitively influence walking, novices did not receive explicit instructions or training on how to walk and were given only one or two practice trials walking solo and with a partner.

#### Solo walking condition

The first condition required the novice to walk forward alone to provide data on their solo preferred gait parameters. Four trials were performed in one block.

#### Partnered backleading conditions

Next, the expert influenced changes to the novice’s step frequency in three partnered backleading conditions—Decrease, Maintain, or Increase step frequency relative to the novice’s Solo preferred step frequency. The Maintain condition was designed to result in similar gait kinematics as the Solo condition while allowing measurement of hand interaction forces. The expert held the opposite ends of the custom force handles and walked backwards while the novice walked forwards (Fig. 1a).

All backleading conditions occurred in one block. At the beginning of the block, the novice was instructed that “Your partner may nonverbally suggest that you walk in a different way; try to cooperate with your partner.” 8 trials were performed for each backleading condition, with trial order randomized. 5-min seated rest breaks were enforced after every 10 trials.

At the beginning of each trial, the expert received audio instructions on how to influence the novice’s step frequency (Decrease, Maintain, or Increase). The novice was then given an audio cue through the headphones to start walking, and the expert started walking in response to the novice’s gait initiation. After the start cue to the novice, the expert listened to metronome beats occurring at 75, 100, or 125% of the novice’s Solo preferred step frequency, depending on the condition (Decrease, Maintain, or Increase step frequency, respectively).

### Data preprocessing

All motion capture data were labeled and gapfilled in Vicon Nexus and exported to Matlab to be low-pass filtered at 10 Hz with a 4th order Butterworth filter. Velocity data were obtained from the time derivative of marker position data.

Force data were also low-pass filtered at 10 Hz with a 4th order Butterworth filter and then downsampled to match the sampling rate of motion capture data. To account for sensor drift, we subtracted zero-force voltage bias from force data for each partnership. We calculated zero-force voltage bias during the first solo walking trial after the block of partnered backleading conditions, except for one participant who was missing data from this trial, for whom we used the last solo walking trial before the partnered conditions. We analyzed hand interaction forces in the anterior–posterior direction only from the sensor held by the novice’s right hand (and the expert’s left hand) as data appeared similar between both force sensors.

### Outcome metrics

#### Time periods of analysis

As we were interested in steady-state behavior, not gait initiation or termination, we determined the novice’s steady-state walking period for each trial. Heelstrike events were identified from local minima of vertical position of heel markers, except for one participant with poor marker fill, for whom we used local minima of anterior–posterior velocity of ankle markers. Heelstrike events were inspected manually and corrected when necessary. To analyze the same number of steps for each trial, we selected the middle 4 steps starting with the second right heelstrike and ending with the 4th right heelstrike (Fig. 1c, top). To check that the novice maintained constant gait velocity, we tested for a significant linear trend in torso velocity during this period (Fig. 1c, bottom). Outcome metrics were calculated for the trial if either a) there was no significant trend (p > 0.05) or b) there was a significant trend (p < 0.05) and the coefficient corresponding to acceleration was below a threshold of 0.001 m/s^2^.

To remove variability due to the partners resetting their starting positions in the room before each trial, several steady-state metrics were normalized by subtracting the mean from a baseline “pre-move” period when both partners were standing still. The pre-move period for each trial was selected as the 0.49 s time window before the earliest movement onset of either partner, which was determined by when the novice or expert’s torso velocities crossed above a threshold of 0.1 m/s.

#### Novice gait parameters

We calculated several gait parameters during the steady-state period to examine both the intended changes to step frequency and any additional changes potentially due to gait coupling. Step frequency was calculated as the inverse of the elapsed time between successive heelstrike events (Fig. 1c, top). The mean across the middle 4 steps was calculated for each trial. Step length was calculated as the anterior–posterior distance between foot markers at heelstrike events (Fig. 1c, top), and the mean across the middle 4 steps was calculated per trial. Gait speed was calculated as the anterior–posterior displacement of the torso marker divided by time elapsed during the steady-state period. Step frequency, step length, and gait velocity were all normalized to each participant’s means during the Solo walking condition.

Young persons without walking or balance impairments have been shown to maintain a constant “walk ratio” between step length and step frequency across gait speeds during preferred solo walking^20–22^. Thus, we calculated the walk ratio as a metric of gait coupling, using the normalization procedure established in previous literature^20^.

#### Force and power metrics

We analyzed both the signs and magnitudes of force and power to examine whether this data contained information on desired walking behavior and/or mechanically constrained human movement. The mean signed and absolute value of force and power were calculated during the steady-state period for each trial (Fig. 3a,c). To obtain the full range of forces and power used, we calculated histograms including every time sample (after downsampling) of every trial and participant for each backleading condition, using Sturges’ rule^48^ to choose the number of bins.

We calculated mechanical power at both the interaction point between partners and at the novice’s torso to check for any differences between these locations. Mechanical power was calculated as anterior–posterior force multiplied by anterior–posterior velocity of (a) the interaction point marker—chosen as either the marker located slightly proximal of the novice’s right metacarpophalangeal joint (n = 4) or the marker on the novice’s right radial styloid process (n = 6) and (b) the novice’s torso marker. All power metrics were normalized by each participant’s body mass. Power calculations for the two locations resulted in nearly identical values (compare middle and bottom rows in Supplementary Fig. S1 online), so we examined only power at the interaction point for further analyses.

To examine whether hand interactions relied on mechanical effects to alter the novice’s gait, we compared the total power transfered at the hands during backleading to estimated power from the lower limbs for propelling solo walking. Power measured at the hand during backleading was multiplied by two to account for total power from both hands and to compare to power for propelling walking. Whereas interaction forces and power transfer between wearable robotic devices and the human body are difficult to measure^28,29^ and seldom reported^30^, the mechanical power for propelling locomotion can be estimated as the power exerted by human lower limbs on the center-of-mass to maintain constant-speed walking without external aid^23^. The estimated power was calculated using an equation describing power for performing step-to-step transitions as a function of step length during constant-speed solo walking in unimpaired adults^23^. We normalized step lengths during backleading by mean step lengths measured during solo walking for each novice and used these normalized values as inputs to the equation.  We also calculated  changes in power between conditions (i.e., Maintain—Decrease and Increase—Maintain) for the hands during backleading  and for propelling locomotion  for changes in measured step lengths between conditions.

### Spatiotemporal metrics characterizing control strategies

To characterize spatial synchrony between partners, the Inter-torso distance (ITD) was calculated as the anterior–posterior distance between partners’ torsos during the steady-state period (Fig. 5a) and is based on the “whole-body synchronization” metric^9^. Change in ITD was calculated by subtracting the mean ITD during the pre-move period (Fig. 5a) from the mean ITD during steady state for each trial. Change in ITD is negative if partners are closer together and positive if they are further apart during steady-state walking relative to the pre-move period.

To characterize temporal synchrony between partners during steady-state walking, we calculated cross-correlations between anterior–posterior torso velocities (Fig. 5b). Torso velocity during the steady state period was first mean-subtracted, and then cross-correlation was calculated for time delays up to ¼ of the novice’s mean step time—the inverse of mean step frequency—for the trial in order to avoid ambiguity in the signs of cross-correlation and time delay for cyclical signals. The maximum absolute cross-correlation and its corresponding time delay were obtained as the temporal synchrony metrics for each trial.

To determine individual contributions to inter-partner spatial synchrony, the Effective Arm Length (EAL) for each partner was calculated as the anterior–posterior distance between each partner’s torso marker and the interaction point marker (Fig. 5a). Similar to the Change in ITD, Change in EAL was calculated by subtracting the mean EAL during the pre-move period (Fig. 6a) from the mean EAL during steady state for each trial.

To characterize temporal order of movement for each partner’s torso relative to the interaction point during steady-state walking, we performed cross-correlation analysis between torso and interaction point velocities for each partner (Fig. 6b) using the same procedure as for the cross-correlation between partners’ torso velocities.

#### Models of pHHI dynamics

To glean principles from pHHI applicable to pHRI controllers, we modeled the effective dynamics relating hand interaction force to inter-torso or arm kinematics (Fig. 7) using a “template,” defined as “the simplest model (least number of variables and parameters) that exhibits a targeted behavior”^19^. Our lumped-parameter model characterizes the system’s total effective dynamics, which includes the combined effects of closed-loop neural commands, muscle activation dynamics, and mechanical impedance. Our prior study demonstrated that a linear model between whole-body movement and hand interaction force can characterize pHHI to aid balance in the frontal plane^15^, and our current study applies this type of model to characterize pHHI for backleading in the sagittal plane.

Furthermore, our template model examines the effective dynamics of physical interaction separate from the dynamics of the targeted motor behavior. Previous models of pHHI dynamics were developed based on experimental paradigms where both partners were stationary/seated and the site of physical interaction coincided with the site of targeted motor behavior at the interaction point between partners’ hands^49–52^. As such, these models of pHHI assume that both partners intended to control mechanics of a common interaction point as a shared task goal. In contrast, both partners locomote in our backleading paradigm, so we develop novel models that define the task goal as either (a) inter-partner coordination or (b) controlling one’s own arm effective dynamics.

Maintaining a constant distance between partners’ bodies as well as constant individual arm postures have been established as relevant goals for haptic communication during partnered walking^9^. Thus we assumed that interaction forces act to maintain Inter-torso distance (ITD) or Effective arm length (EAL) at reference values measured during the pre-move period. This model assumption reflects task goals of walking synchronously while maintaining the same arm posture as during standing. These models represent lumped effects of various anatomical body segments on the effective inter-torso and arm systems as defined by the ITD and EAL.

To obtain the model coefficients describing inter-torso and arm effective dynamics, we performed multiple linear regression of interaction force data to change in ITD and EAL, respectively, and their first derivatives (i.e. velocity). The regression algorithm for each trial consisted of (1) regressing to both displacement and velocity terms, (2) discarding any non-significant terms, as defined by regression coefficients with confidence intervals that included zero, and repeating steps (1) and (2) until only significant terms or no terms remained in the final regression model. If the final regression model reached statistical significance (p < 0.05), the coefficients from the trial were included in calculations of the mean displacement and velocity coefficients, k_p_ and k_v_. The R^2^ value of the final model was calculated to assess quality of fit for each trial. As tension forces are positive in sign, k_p_ and k_v_ are positive if tension increases as displacement increases, i.e. when forces act to oppose changes in ITD or EAL.

We used the interaction force—measured by the force sensor between the partners—for our arm effective dynamics models as we assume that accelerations of the effective arm system are negligible, and thus all forces on the effective arm sum to zero. This assumption then results in equal forces on the effective arm system from the torso and hands for each partner.

### Statistical analysis

First, to validate Maintain as an appropriate baseline/control condition for metrics measured both during Solo and partnered conditions, we verified that the partnered Maintain condition was not statistically different from the Solo walking condition. We compared paired samples using either a parametric or non-parametric test depending on if data were normally distributed, as determined by the Lilliefors test. If both samples were normally distributed, we used the Student’s *t* test for equal sample sizes or Welch’s *t* test for unequal sample sizes. If either sample was not normally distributed, we used the Wilcoxon sign-rank test for equal sample sizes or Mann–Whitney *U* test (a.k.a. the “rank sum test”) for unequal sample sizes. We found no significant difference (p > 0.05) between Maintain and Solo condition means for step frequency, step length, gait velocity, Change in EAL, time delay between torso velocities, maximum absolute cross-correlation between torso velocities, or mean interaction point velocity. Thus, we used the Maintain condition for all further statistical analysis.

We then performed repeated measures ANOVAs to examine the effect of backleading condition (Decrease, Maintain, Increase) on each outcome metric. First, we performed Mauchly’s test of sphericity. We then calculated the F-value, and if sphericity was violated, we used the Greenhouse–Geisser correction for the degrees of freedom and p-value. We followed up significant ANOVAs with Bonferroni-corrected pairwise comparisons between condition means. An alpha-value of 0.05 was used for all tests of significance.

To examine whether there was matching of effective dynamics between partners, we performed linear correlations on novice and expert arm model coefficients. Correlations between paired samples (novice and expert) of displacement and velocity coefficients were tested separately for each backleading condition. We first used the Lilliefors test to determine if each sample was normally distributed and then Pearson’s correlation or Spearman’s rank correlation for data with normal or non-normal distributions, respectively. An alpha-value of 0.05 was used to test for significant correlations.

### Supplementary Information

Supplementary Figure S1.

## Data Availability

The datasets generated and analyzed during the current study are available in the Open Science Foundation repository: https://osf.io/jyg4r/.
