# Supplementary material for: Low-force human–human hand interactions induce gait changes through sensorimotor engagement instead of direct mechanical effects
Source: Sci Rep. 2024 Feb 13;14:3614. doi: 10.1038/s41598-024-53991-4 (PMC10864400; doi:10.1038/s41598-024-53991-4)
Supplement: Supplementary file 1 — Supplementary Figure S1. [file 41598_2024_53991_MOESM1_ESM.pdf]

# Low-force human-human hand interactions induce gait changes through sensorimotor engagement instead of direct mechanical effects

Mengnan Wu[1*], Madeleine E. Hackney[2,3], Lena H. Ting[1,3]

[1]The Wallace H. Coulter Department of Biomedical Engineering, Emory University and Georgia Institute of Technology, Atlanta, GA, United States

[2]Department of Medicine, Division of Geriatrics and Gerontology, Emory University School of Medicine, Atlanta, GA, United States

[3]Department of Rehabilitation Medicine, Division of Physical Therapy, Emory University School of Medicine, Atlanta, GA, United States

*corresponding author email: mw40@emory.edu

# Supplementary Figure 1

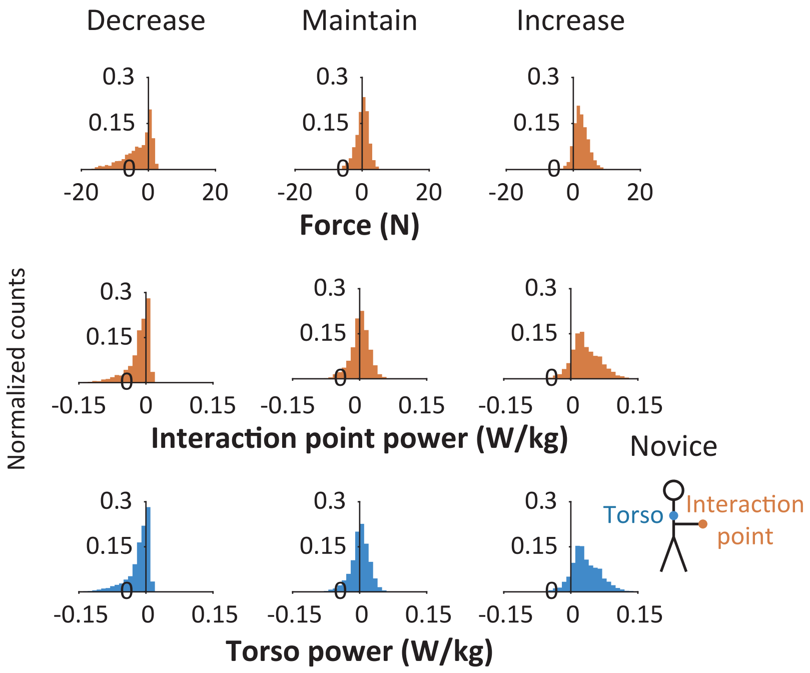


***Supplementary figure S1: Histograms of interaction force, power at the interaction point, and power at the novice's torso.*** *Values calculated at the interaction point are in orange and at the torso in blue. Data from all time samples, trials, and participants (n = 8) are included. Columns correspond to backleading conditions.*
